# A Comparative In Vitro Analysis of Antimicrobial Effectiveness and Compressive Strength of Ginger and Clove-Modified Glass Ionomer Cement

**DOI:** 10.7759/cureus.55964

**Published:** 2024-03-11

**Authors:** Jessy Paulraj, Blessy Pushparathna, Subhabrata Maiti, Neha Sharma, Rajeshkumar Shanmugam

**Affiliations:** 1 Department of Pedodontics and Preventive Dentistry, Saveetha Dental College and Hospitals, Saveetha Institute of Medical and Technical Sciences, Saveetha University, Chennai, IND; 2 Department of Prosthodontics, Saveetha Dental College and Hospitals, Saveetha Institute of Medical and Technical Sciences, Saveetha University, Chennai, IND; 3 Nanobiomedicine Lab, Centre for Global Health Research, Saveetha Dental College and Hospitals, Saveetha Institute of Medical and Technical Sciences, Saveetha University, Chennai, IND

**Keywords:** restorative dentistry, strength, antimicrobial activity, ginger extract, clove, modified glass ionomer cement

## Abstract

Background

Glass ionomer cement (GIC) is widely recognized for its self-adhesive characteristics and biocompatibility, making it commonly used as a restorative material. However, challenges related to limited antibacterial effectiveness and relatively low mechanical properties have hindered its widespread clinical use. Clove and ginger are recognized for their potent antimicrobial activity against numerous pathogenic microorganisms. The present study aims to enhance the clinical applicability of GIC by modifying it with clove and ginger extract.

Aim

The objective of the study is to assess the antimicrobial effectiveness and compressive strength of GIC modified with ginger and clove extract.

Materials and methods

Ginger and clove extracts were prepared and incorporated into conventional GIC at three concentrations for each, creating ginger-modified GIC groups (Group A, Group B, and Group C) and clove-modified GIC groups (Group D, Group E, and Group F), with Group G as the control (conventional GIC without modification). The antimicrobial assessment was conducted on disc-shaped GIC specimens (3.0 mm height x 6.0 mm diameter) prepared using molds. Bacterial strains were used to evaluate antimicrobial properties, with minimum inhibitory concentration (MIC) assays conducted at intervals of one to four hours for both modified and unmodified groups. Compressive strength specimens were prepared using cylindrical molds (6.0 mm height × 4.0 mm diameter), according to the ISO (International Organization for Standardization) guidelines. The evaluation was conducted using a Zwick universal testing machine (ElectroPuls® E3000, Instron, Bangalore, India), with the highest force at the point of specimen fracture recorded to determine compressive strength. Statistical analysis was conducted utilizing a one-way analysis of variance (ANOVA) alongside Tukey’s post hoc test, with a significance threshold set at p < 0.01.

Results

The antimicrobial effectiveness of clove and ginger-modified GIC was assessed through a MIC assay, revealing a statistically significant improvement in antimicrobial potency against *Streptococcus mutans* and *Lactobacillus* within the modified groups compared to the control group (p < 0.01). Increased extract concentration correlated with enhanced antimicrobial activity. Clove-modified GIC exhibited superior antimicrobial efficacy compared to ginger extract. Compressive strength was higher in clove-modified GIC groups (p < 0.01), with Group F showing a maximum value of 175.88 MPa, while other modified groups demonstrated similar results to the control, with a value of 166.81 MPa (p > 0.01).

Conclusion

The study concludes that both clove-modified GIC and ginger-modified GIC exhibited antimicrobial activity against *Streptococcus mutans* and *Lactobacillus* species. The antimicrobial activity was notably higher in clove-modified GIC compared to ginger-modified GIC. Additionally, the compressive strength of clove-modified GIC surpassed all other groups. Thus, clove-modified GIC emerges as a promising restorative material for addressing recurrent caries. Future investigation is necessary to assess the long-term durability of the material.

## Introduction

Glass ionomer cement (GIC) is commonly used in dental restorations due to its ease of handling, self-setting properties, and adhesion [1]. However, its mechanical strength limitations and its ability to effectively inhibit a broad spectrum of bacteria are questionable, which raises concerns about its long-term performance and effectiveness in preventing secondary caries [1]. Prior studies have assessed the clinical effectiveness of various conventional restorative materials in posterior primary teeth, revealing a higher risk of failure for restorations in primary molars, likely attributed to drawbacks such as low wear resistance and inferior compressive strength [2]. To mitigate the occurrence of secondary caries and enhance the strength of these materials, various studies have incorporated antimicrobial compounds and nanoparticles into GIC formulations to improve their overall properties [2]. In pursuit of enhancing desired properties, GICs have been integrated into various biomaterials endowed with antibacterial capabilities. The initial adjustments effectively enhanced the mechanical characteristics of the material without causing any detrimental effects [3]. However, careful consideration is essential in the selection of these substances to ensure they are non-toxic to pulp or gingival cells while effectively guarding against the proliferation of cariogenic bacteria [4]. Centuries ago, medicinal plants were extensively utilized for therapeutic purposes and prevention of illnesses, offering relief or cure. Phytomedicine, which utilizes various plant parts or extracts for therapeutic or health-enhancing purposes, has shown effectiveness. The use of herbal extracts offers the advantage of producing positive effects without the potential development of bacterial resistance [5]. Notably, the World Health Organization reports that a substantial 80% of the global population relies on traditional herbal medicine to address their primary healthcare needs. Utilizing herbs and herbal extracts stands out as a natural and secure method to reduce toxicity.

Ginger (*Zingiber officinale*) is a flowering plant valued for both its culinary and medicinal properties. The rhizome, an underground stem part, is widely used as a spice, acknowledged for its health benefits, encompassing pharmacological effects, antioxidant properties, antibacterial attributes, anti-inflammatory actions, antinociceptive effects, and anti-mutagenic attributes. The ginger root, belonging to the *Zingiberaceae* family, contains more than 1200 species in 53 genera. With its diverse applications, including traditional medicine, ginger has gained popularity globally. Notably, the phenolic and terpene molecules, particularly parasols, shogaols, and gingerols, contribute to its medicinal properties. Gingerols are the predominant phenolic compounds in fresh ginger, known for their various health-promoting effects and antibacterial properties [6,7].

Cloves (*Syzygium aromaticum*), derived from the aromatic flower buds of a *Myrtaceae* family tree, are utilized for their essential oil, which acts as a pain reliever for dental emergencies. With a history spanning over 2,000 years in India and China, cloves have been utilized for their spice properties, combating tooth decay, and addressing bad breath [6]. Notably, cloves demonstrate efficacy against bacteria linked to dental issues, periodontal disease, and other microbial threats. Eugenol, a key component in cloves, plays a significant role in battling oral microbes and is integrated into dental restorative materials. Researchers are exploring cloves as a natural means to maintain dental health due to their impact on plaque, gingivitis, and oral flora, and studies have also indicated that clove oil exhibits potent antibacterial action [8]. In the context of the information, the present research aimed to evaluate the antibacterial effectiveness and compressive strength of GIC altered with extracts from *Zingiber officinale* (ginger) and *Syzygium aromaticum* (clove). The null hypothesis posited that these extracts would not exhibit antibacterial effects or influence the compressive strength in comparison to conventional GIC.

## Materials and methods

Study design, ethical approval, estimation of sample size, and materials used

In this in vitro study, ethical approval was obtained from the institutional review board under the reference number SRB/SDC/UG-1858/22/PEDO/027. The sample size calculation was conducted using the G*Power sample power calculator, indicating that for a sample power of 0.95 (95% confidence interval) and an effect size of 0.25, each parameter (antimicrobial activity and compressive strength) would need to include 84 samples. The study involved the assessment of antimicrobial efficacy and compressive strength using the following materials: (a) bacterial strains, including *Streptococcus mutans* and *Lactobacillus*; (b) ginger rhizomes; (c) clove buds; and (d) type II conventional GIC from GC Corporation (Tokyo, Japan).

Preparation of extracts

**Figure 1:**
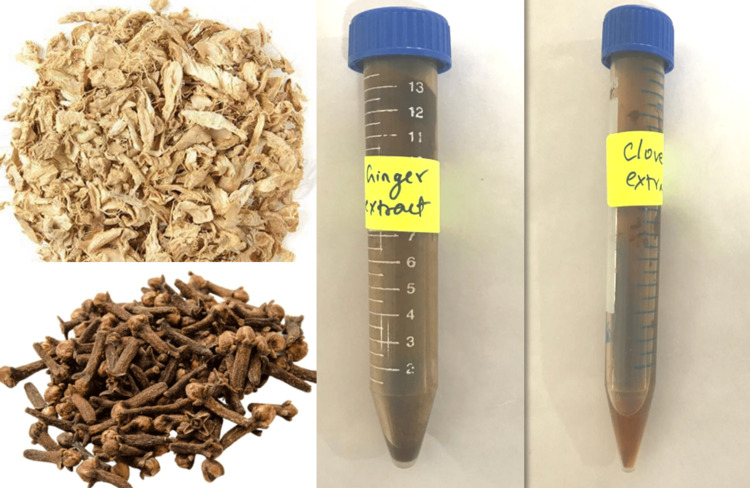
Ginger and clove extract

Clove buds and ginger rhizomes, both dried for five days, were utilized in the experiment. Glassware was cleaned, rinsed with distilled water, and dried at 70°C. Cloves (1 g) were mixed with 100 mL distilled water and heated using a heating mantle at 60-70°C for 15 minutes. After filtration through Whatman No. 1 filter paper (Whatman Plc, Maidstone, UK), 80 ml of the filtrate was gathered into a separate Erlenmeyer flask. This filtered extract was then condensed to a volume of 5 ml. The same procedure was applied to ginger extract (Figure 1).

Preparation of GIC-modified specimens

**Figure 2:**
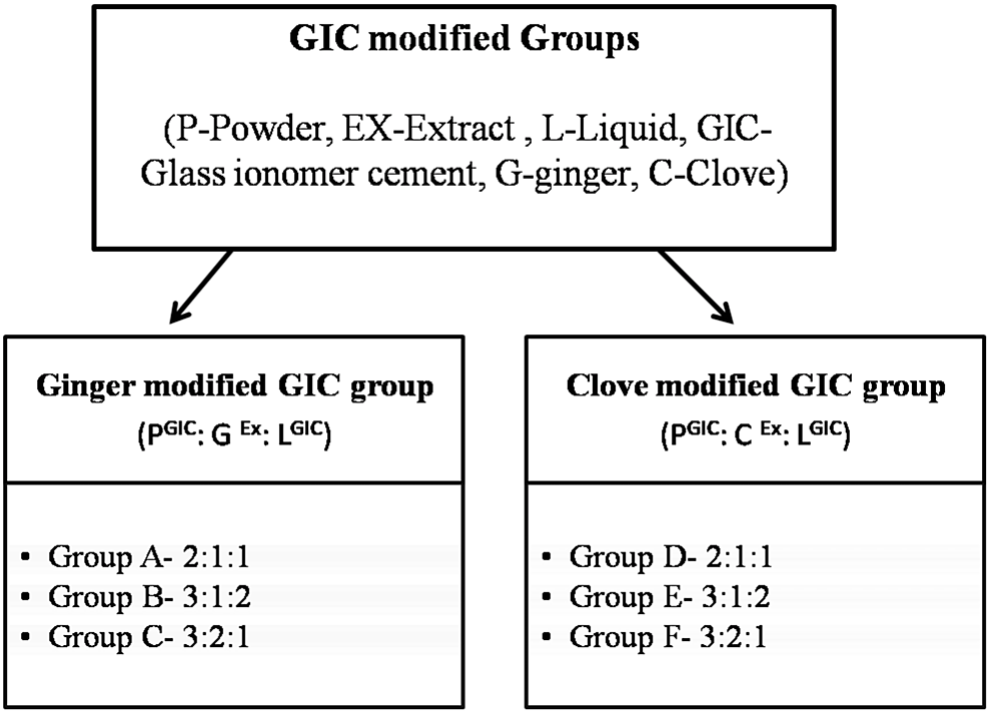
Proportion of GIC-modified groups GIC: glass ionomer cement.

Ginger and clove extracts were mixed with conventional GIC in three different concentrations (Figure 2).

**Figure 3:**
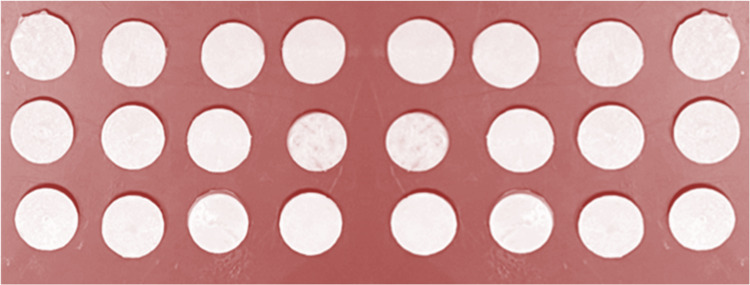
Test samples

A total of 84 disc-shaped GIC specimens were fabricated by initially blending conventional GIC components with the extract. The resulting cement was poured into split Teflon molds measuring 3 mm in height and 6 mm in diameter. After setting, the samples were extracted from the molds for antibacterial activity assessment (Figure 3).

Following the manufacturer's instructions, the specimens were solidified for 20 minutes and sterilized with ultraviolet UV radiation for 30 minutes. Each group consisted of 12 specimens, divided evenly between testing against *Streptococcus mutans* and *Lactobacillus*, resulting in six specimens for each bacterial strain. Compressive strength was evaluated as per ISO (International Organization for Standardization) 9917-1:2007 using cylindrical molds (4.0 mm diameter × 6.0 mm height), with each group containing 12 specimens, making a total of 84 specimens overall. The materials were placed in the molds, leveled, and allowed to set for 20 minutes.

Determination of antimicrobial activity

**Figure 4:**
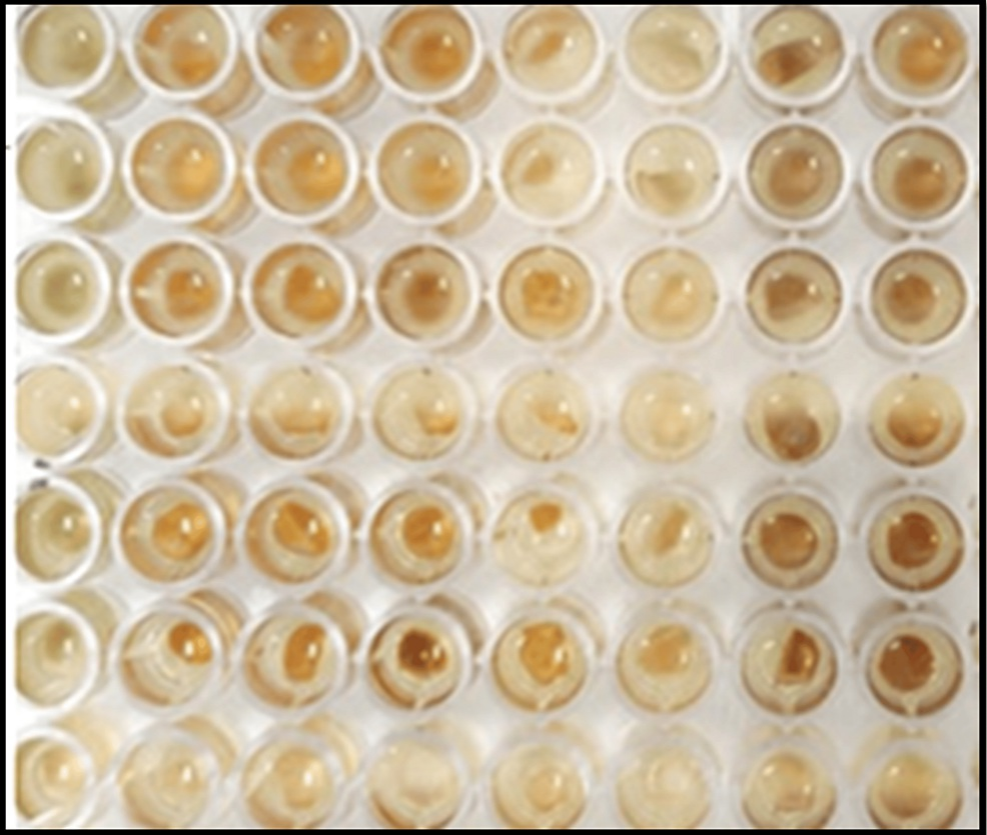
MIC assay in ELISA 96-well plate MIC: minimum inhibitory concentration; ELISA: enzyme-linked immunosorbent assay.

The isolated bacterial (*Streptococcus mutans* and *Lactobacillus acidophilus*) strains were obtained from the Department of Microbiology and cultured on Mueller Hinton agar. After subculturing, they were inoculated in Mueller Hinton broth and incubated at 37°C for 24 hours. The suspension was adjusted, and the minimum inhibitory concentration (MIC) was determined. Each dilution (200 µl) was added to enzyme-linked immunosorbent assay (ELISA) plates with agar seeded (50 µl) with the test strain (Figure 4).

Twelve specimens from each group were incubated at 37°C for various intervals, ranging from the first to the fourth hour, and cell viability was assessed at regular intervals by measuring the absorbance at 540 nm using an ELISA reader.

Compressive strength measurement

**Figure 5:**
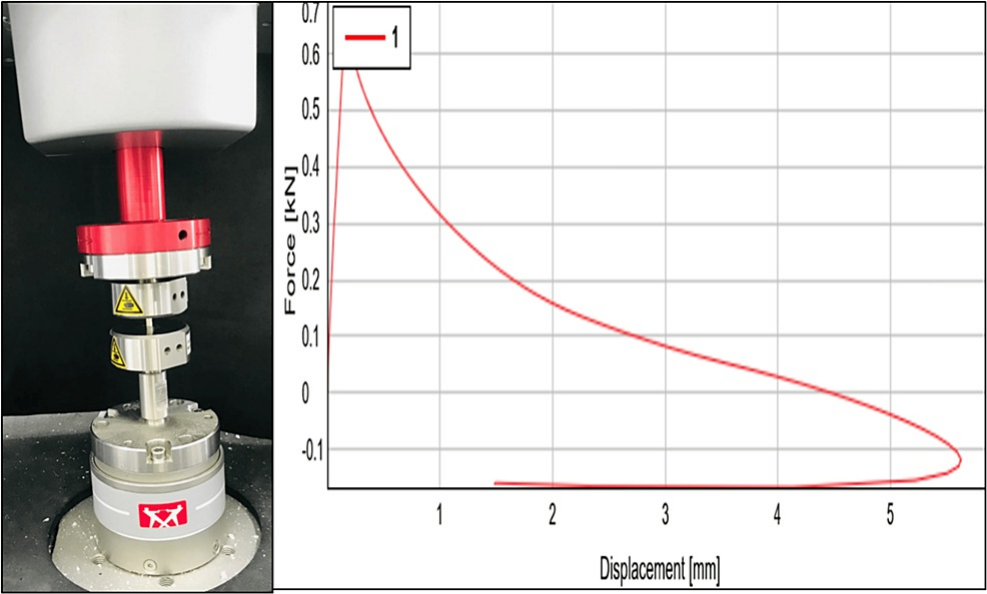
Compressive strength testing

After demolding, specimens were stored in water for 24 hours before testing with a Zwick universal testing machine (ElectroPuls®, Instron, Bangalore, India) at a crosshead speed of 0.5 mm/min, conducted 24 hours after mixing. The maximum force at specimen fracture was recorded in MPa to determine compressive strength (Figure 5).

Statistical analysis

Mean differences were statistically analyzed using one-way analysis of variance (ANOVA) followed by Tukey’s post hoc test. This analysis was conducted using Statistical Package for Social Sciences (SPSS) version 24 (IBM Corp., Armonk, NY), with a significance level set at p < 0.01.

## Results

Antimicrobial activity against *Streptococcus mutans*

**Figure 6:**
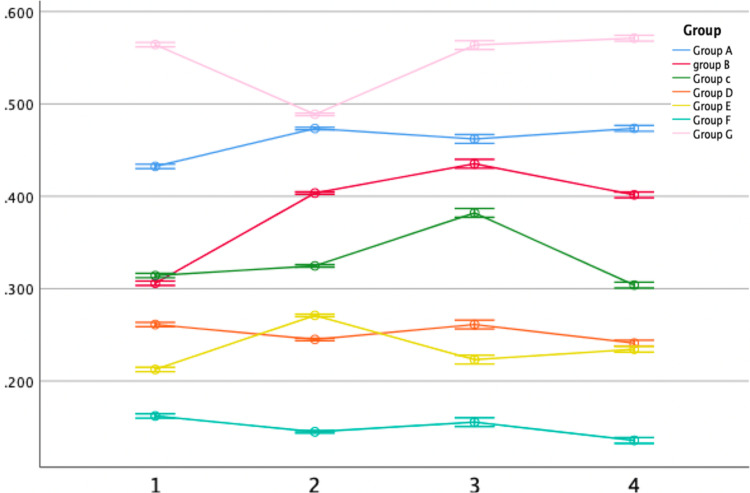
Antimicrobial efficacy against Streptococcus mutans The X-axis represents the time interval (hours) and the Y-axis represents the minimum inhibitory concentration (MIC) mean value.

Regarding *Streptococcus mutans*, the modified groups exhibited superior performance compared to the control (Group G), showing significant differences. The concentration ratio of 3:2:1 (Group F) demonstrated strong antimicrobial activity (Figure 6), confirming that a higher concentration led to enhanced antimicrobial activity. One-way ANOVA analysis revealed a significant difference in antibacterial activity against *Streptococcus mutans* across the first, second, third, and fourth-hour intervals, with Group F consistently exhibiting the lowest mean values (0.162, 0.145, 0.155, and 0.135, respectively), indicating superior performance. When comparing the ginger-modified GIC groups (Groups A, B, and C) with the clove-modified GIC groups (Groups D, E, and F), the clove-modified groups consistently displayed the lowest mean values across all four time intervals, signifying a significant difference. Conversely, the control group (Group G) exhibited higher mean values of 0.564, 0.488, 0.563, and 0.571 in all time intervals, suggesting elevated bacterial growth and reduced antibacterial activity compared to the modified GIC groups (Table 1).

**Table 1:** Comparison of MIC mean value for Streptococcus mutans based on different time intervals * Significant at 0.01, p-value derived from one-way ANOVA. MIC: minimum inhibitory concentration.

Time intervals (hours)	Groups	N	Mean ± std. deviation	Std. error	95% confidence interval for mean		F-value	P-value
					Lower bound and upper bound			
First hour	Group A	6	0.432 ± 0.0013	0.0005	0.430	0.433	13997.27	0.001*
	Group B	6	0.305 ± 0.0057	0.0023	0.299	0.311		
	Group C	6	0.3141 ± 0.0013	0.0005	0.312	0.315		
	Group D	6	0.261 ± 0.0007	0.0003	0.260	0.261		
	Group E	6	0.212 ± 0.0038	0.0015	0.208	0.216		
	Group F	6	0.162 ± 0.0019	0.0007	0.160	0.164		
	Group G (control)	6	0.564 ± 0.0007	0.0003	0.563	0.564		
Second hour	Group A	6	0.473 ± 0.0022	0.0009	0.470	0.475	36659.78	0.001*
	Group B	6	0.403 ± 0.0028	0.0011	0.400	0.406		
	Group C	6	0.324 ± 0.0019	0.0008	0.322	0.326		
	Group D	6	0.245 ± 0.0006	0.0002	0.244	0.245		
	Group E	6	0.271 ± 0.0006	0.0002	0.270	0.271		
	Group F	6	0.145 ± 0.0006	0.0002	0.144	0.145		
	Group G (control)	6	0.488 ± 0.0005	0.0002	0.488	0.489		
Third hour	Group A	6	0.462 ± 0.0008	0.0003	0.461	0.462	3868.23	0.001*
	Group B	6	0.435 ± 0.0011	0.0004	0.433	0.436		
	Group C	6	0.382 ± 0.0008	0.0003	0.381	0.382		
	Group D	6	0.261 ± 0.0007	0.0003	0.260	0.261		
	Group E	6	0.223 ± 0.0043	0.0017	0.218	0.227		
	Group F	6	0.155 ± 0.0144	0.0059	0.140	0.170		
	Group G (control)	6	0.563 ± 0.0005	0.0002	0.563	0.564		
Fourth hour	Group A	6	0.473 ± 0.0035	0.0014	0.469	0.477	9744.22	0.001*
	Group B	6	0.401 ± 0.0010	0.0004	0.400	0.402		
	Group C	6	0.303 ± 0.0011	0.0004	0.302	0.305		
	Group D	6	0.241 ± 0.0008	0.0003	0.240	0.241		
	Group E	6	0.234 ± 0.0012	0.0004	0.233	0.235		
	Group F	6	0.135 ± 0.0090	0.0036	0.126	0.145		
	Group G (control)	6	0.571 ± 0.0009	0.0004	0.570	0.572		

Furthermore, Tukey's honestly significant difference (HSD) test revealed a statistically significant difference between the control group and the other groups (p < 0.01), where the control group (Group G) performed the least. A statistically significant difference was noticed when comparing groups D, E, and F with the other groups, proving that the clove-modified groups (Group D, E, and F) exhibited high antimicrobial activity (p < 0.01) compared to the ginger-modified groups (Group A, B, and C) (Table 2).

**Table 2:** Pairwise comparison of antimicrobial efficacy on Streptococcus mutans between all groups The error term is mean square (error) = 3.054E-6. * Significant at the 0.01 level; the p-value was derived from multiple comparisons by Tukey's honestly significant difference (HSD).

Pairwise comparison	Mean difference	Sig.	95% confidence interval	
			Lower bound	Upper bound
Group A vs Group B	0.073	0.001*	0.070	0.076
Group A vs Group C	0.129	0.001*	0.125	0.132
Group A vs Group D	0.208	0.001*	0.205	0.211
Group A vs Group E	0.225	0.001*	0.221	0.228
Group A vs Group F	0.310	0.001*	0.307	0.313
Group A vs Group G	-0.086	0.001*	-0.089	-0.083
Group B vs Group C	0.055	0.001*	0.052	0.058
Group B vs Group D	0.134	0.001*	0.131	0.137
Group B vs Group E	0.151	0.001*	0.148	0.154
Group B vs Group F	0.236	0.001*	0.233	0.240
Group B vs Group G	-0.160	0.001*	-0.163	-0.157
Group C vs Group D	0.079	0.001*	0.075	0.082
Group C vs Group E	0.095	0.001*	0.092	0.099
Group C vs Group F	0.181	0.001*	0.178	0.184
Group C vs Group G	-0.215	0.001*	-0.218	-0.212
Group D vs Group E	0.016	0.001*	0.013	0.019
Group D vs Group F	0.102	0.001*	0.099	0.105
Group D vs Group G	-0.294	0.001*	-0.297	-0.291
Group E vs Group F	0.085	0.001*	0.082	0.088
Group E vs Group G	-0.311	0.001*	-0.314	-0.308
Group F vs Group G	-0.397	0.001*	-0.400	-0.394

Antimicrobial activity against *Lactobacillus*

**Figure 7:**
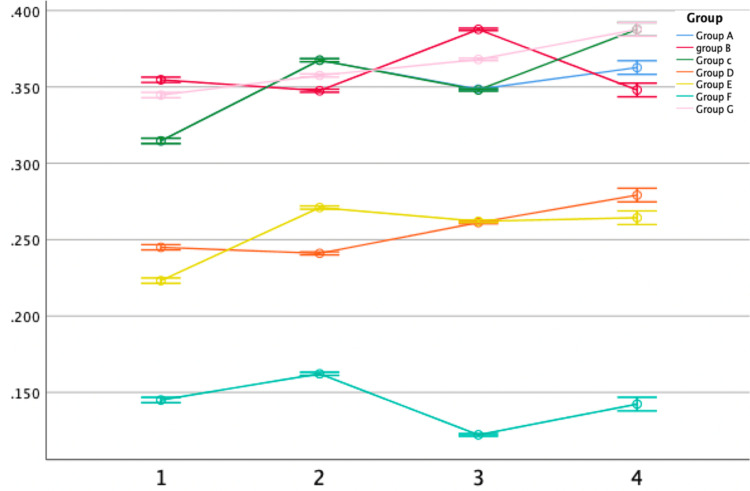
Antimicrobial efficacy against Lactobacillus The X-axis represents the time interval (hours) and the Y-axis represents the minimum inhibitory concentration (MIC) mean value.

Noticeable variations in antimicrobial activity against *Lactobacillus* were observed between the modified and control groups (Group G). The linear graph from the repeated measure ANOVA revealed that clove-modified GIC groups (Groups D, E, and F) displayed higher antibacterial activity against *Lactobacillus* than the other groups (Figure 7).

The one-way ANOVA analysis revealed significant differences across the first, second, third, and fourth-hour intervals against *Lactobacillus*, with Group F consistently exhibiting the lowest mean values of 0.145, 0.162, 0.122, and 0.142, respectively, suggesting superior antibacterial activity. When comparing the ginger-modified (Groups A, B, and C) and clove-modified (Groups D, E, and F) GIC groups, the clove-modified groups consistently displayed lower mean values across all time intervals, indicating significant distinction and demonstrating robust antimicrobial activity (Table 3).

**Table 3:** Comparison of MIC mean value for Lactobacillus based on different time intervals * Significant at 0.001, p-value derived from one-way ANOVA. MIC: minimum inhibitory concentration.

Time interval (hours)	Groups	N	Mean ± std. deviation	Std. error	95% confidence interval for mean		F-value	P-value
					Lower bound	Upper bound		
First hour	Group A	6	0.314 ± 0.00163	0.0006	0.312	0.316	8002.84	0.001*
	Group B	6	0.354 ± 0.00163	0.0006	0.352	0.356		
	Group C	6	0.314 ± 0.00163	0.0006	0.312	0.316		
	Group D	6	0.245 ± 0.00063	0.0002	0.244	0.245		
	Group E	6	0.223 ± 0.00435	0.0017	0.218	0.227		
	Group F	6	0.145 ± 0.00063	0.0002	0.144	0.145		
	Group G (control)	6	0.344 ± 0.00163	0.0006	0.342	0.346		
Second hour	Group A	6	0.367 ± 0.00104	0.0004	0.366	0.368	25151.21	0.001*
	Group B	6	0.347 ± 0.00122	0.0005	0.346	0.348		
	Group C	6	0.367 ± 0.00122	0.0005	0.366	0.368		
	Group D	6	0.241 ± 0.00089	0.0003	0.240	0.241		
	Group E	6	0.271 ± 0.00063	0.0002	0.270	0.271		
	Group F	6	0.162 ± 0.00194	0.0007	0.160	0.164		
	Group G (control)	6	0.357 ± 0.00122	0.0005	0.356	0.358		
Third hour	Group A	6	0.348 ± 0.00054	0.0002	0.347	0.349	59146.01	0.001*
	Group B	6	0.387 ± 0.00081	0.0003	0.386	0.388		
	Group C	6	0.348 ± 0.00063	0.0002	0.347	0.348		
	Group D	6	0.261 ± 0.00075	0.0003	0.260	0.261		
	Group E	6	0.262 ± 0.00001	0.0001	0.262	0.262		
	Group F	6	0.122 ± 0.00194	0.0007	0.120	0.124		
	Group G (control)	6	0.368 ± 0.00063	0.0002	0.367	0.368		
Fourth hour	Group A	6	0.362 ± 0.01194	0.0048	0.350	0.375	1628.29	0.001*
	Group B	6	0.348 ± 0.00063	0.0002	0.347	0.348		
	Group C	6	0.387 ± 0.00081	0.0003	0.386	0.388		
	Group D	6	0.279 ± 0.00749	0.0030	0.271	0.287		
	Group E	6	0.264 ± 0.00121	0.0004	0.263	0.265		
	Group F	6	0.142 ± 0.00136	0.0005	0.140	0.143		
	Group G (control)	6	0.387 ± 0.00081	0.0003	0.386	0.388		

Significant differences were observed in pairwise comparisons between the control group and the other groups (p < 0.01), with the control group (Group G) performing the least. Clove-modified GIC groups exhibited superior antimicrobial efficacy compared to ginger-modified groups. Group D and Group E showed similar results against *Lactobacillus*, with no significant difference (p = 0.612, p > 0.01) (Table 4).

**Table 4:** Pairwise comparison of antimicrobial efficacy on Lactobacillus between all groups The error term is mean square (error) = 2.171E-6. * Significant at the 0.01 level; the p-value was derived from multiple comparisons by Tukey's honestly significant difference (HSD).

Pairwise comparison	Mean difference	Sig.	95% confidence interval	
			Lower bound	Upper bound
Group A vs Group B	0.011	0.001*	-0.013	-0.008
Group A vs Group C	0.006	0.001*	-0.008	-0.003
Group A vs Group D	0.091	0.001*	0.089	0.094
Group A vs Group E	0.093	0.001*	0.090	0.095
Group A vs Group F	0.205	0.001*	0.202	0.208
Group A vs Group G	0.016	0.001*	-0.018	-0.013
Group B vs Group C	0.005	0.001*	0.002	0.007
Group B vs Group D	0.102	0.001*	0.100	0.105
Group B vs Group E	0.104	0.001*	0.101	0.106
Group B vs Group F	0.216	0.001*	0.213	0.219
Group B vs Group G	0.005	0.001*	-0.007	-0.002
Group C vs Group D	0.097	0.001*	0.095	0.100
Group C vs Group E	0.099	0.001*	0.096	0.101
Group C vs Group F	0.211	0.001*	0.208	0.214
Group C vs Group G	0.010	0.001*	-0.012	-0.007
Group D vs Group E	0.001	0.612	-0.001	0.004
Group D vs Group F	0.113	0.001*	0.111	0.116
Group D vs Group G	0.107	0.001*	-0.110	-0.105
Group E vs Group F	0.112	0.001*	0.109	0.114
Group E vs Group G	0.109	0.001*	-0.111	-0.106
Group F vs Group G	0.221	0.001*	-0.224	-0.218

Compressive strength

Compressive forces were exerted on the samples, and the resulting linear graph values were examined. Significant differences in compressive strength among the groups were evaluated using a one-way ANOVA, yielding an F-value of 127.2 (Table 5).

**Table 5:** Comparison between groups for evaluation of compressive strength * Significant at 0.01; the p-value was derived by one-way ANOVA.

Groups	n	Mean ± SD	Standard error	Lower bound	Upper bound	F-value	P-value
Group A	12	166.63 ± 0.574	0.165	166.26	166.99	127.2	0.001*
Group B	12	167.133 ± 0.820	0.236	166.61	167.65		
Group C	12	167.100 ± 0.751	0.217	166.62	167.57		
Group D	12	172.791 ± 2.265	0.654	171.35	174.23		
Group E	12	172.125 ± 1.078	0.311	171.43	172.81		
Group F	12	175.883 ± 0.985	0.284	175.25	176.50		
Group G (control)	12	166.816 ± 0.693	0.200	166.37	167.25		

Upon pairwise comparison, it was evident that the clove-modified GIC groups (Groups D, E, and F) displayed greater compressive strength compared to the ginger-modified groups and the control (Group G), with statistical significance (p < 0.01). This indicates that the ginger-modified groups (Groups A, B, and C) and the control group (Group G) yielded similar results without significant differences (p > 0.01) (Table 6).

**Table 6:** Pairwise comparison for the evaluation of compressive strength * Significant difference at 0.01; the p-value was derived from Tukey's post hoc test.

Pairwise comparison	Mean difference	Sig	95% confidence interval	
			Lower bound	Upper bound
Group A vs Group B	-0.500	0.937	-1.926	0.926
Group A vs Group C	-0.466	0.955	-1.892	0.959
Group A vs Group D	-6.158	0.001*	-7.584	-4.732
Group A vs Group E	-5.491	0.001*	-6.917	-4.065
Group A vs Group F	-9.250	0.001*	-10.676	-7.823
Group A vs Group G	-0.183	1.000	-1.609	1.242
Group B vs Group C	0.033	1.000	-1.392	1.459
Group B vs Group D	-5.658	0.001*	-7.084	-4.232
Group B vs Group E	-4.991	0.001*	-6.417	-3.565
Group B vs Group F	-8.750	0.001*	-10.176	-7.323
Group B vs Group G	0.316	0.994	-1.109	1.742
Group C vs Group D	-5.691	0.001*	-7.117	-4.265
Group C vs Group E	-5.025	0.001*	-6.451	-3.598
Group C vs Group F	-8.783	0.001*	-10.209	-7.357
Group C vs Group G	0.283	0.997	-1.142	1.709
Group D vs Group E	0.666	0.792	-0.759	2.092
Group D vs Group F	-3.091	0.001*	-4.517	-1.665
Group D vs Group G	5.975	0.001*	4.548	7.401
Group E vs Group F	-3.758	0.001*	-5.184	-2.332
Group E vs Group G	5.308	0.001*	3.882	6.734
Group F vs Group G	9.066	0.001*	7.640	10.492

Furthermore, no significant difference was observed between Group D and Group E (p-value = 0.792), indicating their similar performance. Among all groups, Group F exhibited the highest compressive strength (175.88 MPa), affirming that a higher concentration resulted in enhanced strength.

## Discussion

Dental caries is a prevalent oral condition caused by microbial infections, particularly cariogenic bacteria, with *Streptococcus mutans* being a major contributor. *Streptococcus mutans* produces the enzyme glucosyltransferase, facilitating the formation of water-insoluble glucan from sucrose, which adheres to tooth enamel. This adhesive layer promotes the colonization of *Streptococcus mutans* and other *Streptococci* species. Subsequently, acidogenic bacteria like *Lactobacillus* species generate an acidic environment through sucrose fermentation [9]. The combination of acidogenic and aciduric bacteria leads to the formation of dental plaque, causing localized tooth damage through acid accumulation and mineral decalcification. Understanding the antibacterial activity of newly synthesized restorative materials against these primary pathogens is essential for addressing caries initiation and propagation. The escalating concern of antibiotic resistance in oral pathogens has led to a growing interest in plant-derived therapies, offering the benefits of no side effects and potential long-term use in oral health. The utilization of herbal plant extracts with antibacterial properties has been a traditional approach to combating tooth caries. Ginger (*Zingiber officinale*) is a medicinal plant widely used in global traditional medicine, including Indian Ayurvedic practices. Particularly, the rhizome part of ginger is employed for its antimicrobial and therapeutic effects [10]. Cloves (*Syzygium aromaticum*), recognized as a highly valuable spice, have been used for many centuries as both a food preservative and for numerous medicinal applications. Cloves demonstrate effectiveness against bacteria associated with dental caries, periodontal disease, and a diverse range of other bacteria [6].

Premkishore et al. [11] and Saleh et al. [12] reported the robust antibacterial activity of the ginger extract against *Streptococcus mutans*. Additionally, Hegde et al. [13] conducted a study that established the effective antimicrobial activity of ginger against both *Streptococcus* and *Lactobacillus* species. Furthermore, Ahmed et al. and Azizi et al. demonstrated noteworthy antibacterial activity against *Streptococcus mutans*, *Staphylococcus* spp., *Lactobacillus* spp., and *Streptococcus sanguinis* cariogenic microorganisms [14]. In 2022, Ashour et al. revealed that combining GIC with active components of ginger extract and chlorhexidine could enhance antimicrobial properties effectively [15], a finding consistent with our current study. The current study's results indicate that the antibacterial efficacy of ginger against *Streptococcus mutans* may stem from its valuable phytoconstituents, which exhibit a high affinity for the surface protein antigen of *Streptococcus mutans*. This is further enhanced by hydrophobic interactions with the active site of the protein, amplifying the inhibitory effect against the surface protein antigen of *Streptococcus mutans* [16]. Furthermore, key components found in the ginger extract, including quinones, alkaloids, saponins, flavonoids, glycosides, tannins, and terpenoids, play a major role in its antibacterial properties [17].

Clove has demonstrated antibacterial efficacy against prevalent oral pathogens, including *Streptococcus mutans*, attributed to its disruptive impact on bacterial cell membranes [18]. The study by Voleti et al. affirmed clove oil's antimicrobial activity against *Streptococcus salivarius*, *Streptococcus sanguis*, and *Lactobacillus acidophilus* [19]. In the research conducted by Aneja and Joshi, clove and clove bud oil were identified as potential antimicrobial agents for treating dental caries [20]. Gupta et al.'s comparative study of clove oil and clove extract revealed a higher zone of inhibition in clove oil, contrasting with our findings. This disparity may be attributed to the use of clove extract in our study as clove oil is incapable of chemically adhering to the polyalkenoate matrix and glass may interfere with the material's setting process [21]. Elgamily et al. suggested that clove possesses significant antimicrobial activity against *Streptococcus mutans* and *Lactobacillus acidophilus*, presenting a promising avenue for minimally invasive and adhesive dentistry [22]. The antibacterial properties of cloves are attributed to phytochemical compounds like flavonoids, saponins, tannins, triterpenoids, and steroids, including phytosterol. The current results suggest that both clove and ginger extracts demonstrate antimicrobial properties against *Streptococcus mutans* and *Lactobacillus*. Notably, the antimicrobial activity is significantly more pronounced in clove extract compared to ginger extract, aligning with the observations in a study by Sharma et al. [23], where the extract was utilized without modification. While these extracts have been explored in previous literature for toothpaste, irrigation, and ointments for toothache, this study represents the first attempt to incorporate them into a restorative material. In this study, herbal extracts were directly incorporated into GIC instead of utilizing nanoparticles, taking advantage of their natural sources and bioactive compounds with therapeutic properties. This direct incorporation enhances biocompatibility, reducing the risk of adverse reactions or cytotoxicity compared to synthetic additives or nanoparticles [24]. Additionally, highlighting the specific therapeutic properties of the chosen plant extract emphasizes the impact of this approach over nanoparticle incorporation. A study by Singer et al. also incorporated plant extracts directly into the material, recognizing different plant parts or extracts as therapeutic or health-promoting agents beneficially without the risk of developing bacterial resistance [25]. Within the scope of this investigation, it is deduced that clove-modified GIC demonstrates superior antibacterial activity against *Streptococcus* and *Lactobacillus* species when compared to ginger-modified GIC. The material with the highest extract concentration exhibits significantly heightened activity against *Streptococcus* and *Lactobacillus* species. Moreover, all modified groups exhibit superior antimicrobial activity compared to the conventional group.

According to ISO 9917 (2007), it is imperative to evaluate the compressive strength of dental materials. In the present study, pairwise comparison analysis revealed no significant difference among the ginger-modified groups when compared to the control (Group G), aligning with the findings of Devi et al. [26], Jaidka et al. [27], and Pavithra et al. [28]. These investigations suggested that adding antimicrobial agents at certain concentrations did not adversely affect the compressive strength characteristics of GIC. The clove-modified group exhibited the highest compressive strength when compared to other groups, indicating a significant result. Group F showed pronounced compressive strength when compared to all other groups. This elevated compressive strength in Group F (clove-modified GIC) could be attributed to the higher concentration of clove extract, supported by the phytochemical constituents that enhance compressive strength, which is in alignment with the study done by Singer et al. [25] where the higher concentration of plant extract when incorporated with GIC gave a good compressive strength.

Thus the heightened antibacterial activity and compressive strength of clove-modified GIC is particularly beneficial for patients at high risk of caries and in preventing secondary caries, thus enhancing its clinical efficacy. An inherent limitation of this study is the lack of consideration for intraoral variables such as masticatory stresses, moisture levels, and potential operator discrepancies. However, further investigations in molecular chemistry and clinical settings are recommended to evaluate practical and economic feasibility and endorse its use in clinical applications.

## Conclusions

In conclusion, the incorporation of clove extract into GIC has demonstrated a substantial enhancement in antibacterial activity against caries-promoting organisms and a significant increase in compressive strength, suggesting that clove modification holds promise as an innovative approach to address the limitations associated with conventional GIC. Moreover, ginger-modified GIC exhibited comparable compressive strength results to the control, further highlighting the potential of clove-modified GIC as a viable restorative material for dental applications. Additional research and clinical trials are warranted to validate and explore the long-term stability and clinical applicability of this modified GIC in preventing and combating dental caries.
